# EPSDNet: Efficient Campus Parking Space Detection via Convolutional Neural Networks and Vehicle Image Recognition for Intelligent Human–Computer Interactions

**DOI:** 10.3390/s22249835

**Published:** 2022-12-14

**Authors:** Qing An, Haojun Wang, Xijiang Chen

**Affiliations:** 1 School of Artificial Intelligence, Wuchang University of Technology, Wuhan 430223, China; 2 School of Safety Science and Emergency Management, Wuhan University of Technology, Wuhan 430070, China

**Keywords:** deep learning, vehicle detection, parking space detection, convolutional neural networks

## Abstract

The parking problem, which is caused by a low parking space utilization ratio, has always plagued drivers. In this work, we proposed an intelligent detection method based on deep learning technology. First, we constructed a TensorFlow deep learning platform for detecting vehicles. Second, the optimal time interval for extracting video stream images was determined in accordance with the judgment time for finding a parking space and the length of time taken by a vehicle from arrival to departure. Finally, the parking space order and number were obtained in accordance with the data layering method and the TimSort algorithm, and parking space vacancy was judged via the indirect Monte Carlo method. To improve the detection accuracy between vehicles and parking spaces, the distance between the vehicles in the training dataset was greater than that of the vehicles observed during detection. A case study verified the reliability of the parking space order and number and the judgment of parking space vacancies.

## 1. Introduction

Parking space detection (PSD) is a fundamental problem in the field of computer vision. Finding an empty parking space is becoming more difficult with increases in the number of urban vehicles. In accordance with statistics, 10% of car drivers in the city need to spend a considerable amount of time looking for empty parking spaces. Therefore, studying the detection of parking spaces [1] and providing parking space information to drivers to help them quickly find an empty parking space are urgent concerns.

PSD technology is divided into two major categories: sensor-based and image-based. The first method requires a large number of sensors to cover the entire parking lot [2]. One sensor can only detect one parking space, and it is susceptible to environmental interference. Simultaneously, deploying and maintaining a large number of sensors is expensive. By contrast, the second method requires only a few inexpensive cameras, and each camera has wide coverage. Meanwhile, nearly all parking lots are already equipped with cameras for security monitoring. Therefore, this method is not only economical but also convenient as it uses existing cameras to detect parking spaces.

Some researchers have studied PSD by using frame images extracted from a video stream. For example, a PSD method was proposed in accordance with vehicle feature point and color histogram classification [3]. This method does not consider whether a vehicle is parked in a parking space. Wang et al. [4] used Sobel edge detection to detect a vehicle in a parking space, and a parking space was considered occupied by a vehicle if the percentage of edge pixels exceeded 5% of the total pixels; otherwise, the parking space was considered available. The disadvantage of this method was the poor identification of parking spaces in outdoor parking lots. In consideration of this issue, a detection method (called ParkLotD) [5] was proposed to improve detection accuracy in outdoor parking spaces; this method uses a classifier based on fuzzy C-means clustering and hyper-parameter tuning. On the basis of a texture feature descriptor [6], Almeida et al. [7] used local binary patterns and local phase quantization to perform parking space detection. Shaaban et al. [8] proposed an individual vehicle detection method by using grayscale images acquired from cameras. This method can detect the vacancy of a parking space under different scenes and weather conditions. The advantage of this method is that the detection system does not require high-quality images; therefore, existing surveillance cameras can be used to detect parking spaces. The disadvantage of this method is that a vehicle-like object in a parking space may be recognized as a vehicle.

At present, some researchers have focused on PSD by using machine learning. In summary, these methods can be classified into three major aspects: object detection-based, image segmentation-based, and marking point regression-based methods. An object detection-based method detects the marking points of a parking space via deep learning [9,10,11]. For example, Xiang et al. [12] proposed a PSD method based on the Haar-AdaBoosting algorithm and a convolutional neural network (CNN). Zhang et al. [13] used You Only Look Once Version 2 (YoloV2) to detect parking spaces by identifying marking points in input parking lot images. Zinelli et al. [14] adopted a faster region-based CNN (Faster-RCNN) to detect parking spaces and carry out the classification of parking space occupation. Suhr et al. [15] proposed an end-to-end single-stage parking detection method that can obtain information about the type of parking space and its occupancy. Image segmentation-based methods use a deep neural network to segment parking spot markings and then use the obtained marking lines to infer the location of a parking spot. For example, Jang et al. [16] conducted PSD by using the semantic segmentation of parking space lines. In accordance with vehicle features [17], a CNN was applied to training parking space images, and then the trained model was used for PSD [18]. In [19], Wu et al. proposed a high fusion convolutional network (HFCN) to segment parking space lines. The limitation of this method is that it cannot be applied for PSD in every parking lot because the image characteristics of each parking lot are different. The detection of parking spaces is easily affected by inter-object occlusion; to address this issue, an inference framework with multiple layers was proposed for PSD [20]. Marking point regression-based methods establish regression models to determine the location of parking spot lines [21]. Li et al. [22] conducted the regression of parking spaces in different directions and classified entrance lines for different types of parking spaces to realize the detection of various parking spaces. Karakaya et al. [23] proposed a method for detecting the occupancy rate of parking spaces by using deep learning; this method runs a cyclic neural network on the embedded system to process parking lot images and to collect information on available parking spaces simply. However, this method is unable to identify the distribution of each parking space. Simultaneously, it can only be applied to the detection of parking spaces with clear lane lines, and it has high requirements for the quality of lane line images [24,25,26].

In consideration of the aforementioned issues, the current study proposes a parking detection method without lane line detection. This method directly uses deep learning to conduct vehicle identification and implements parking detection in accordance with vehicle detection. First, the proposed method determines the optimal time interval for reading the video stream frame images by analyzing the time when a vehicle enters and leaves a parking space. Second, the vacancy of the parking space is judged in accordance with the indirect Monte Carlo method. Third, the detected parking spaces are numbered using the hierarchical model and the TimSort algorithm. Simultaneously, the vehicle detection results can be displayed in top-view form.

## 2. Proposed Method

**Figure 1:**
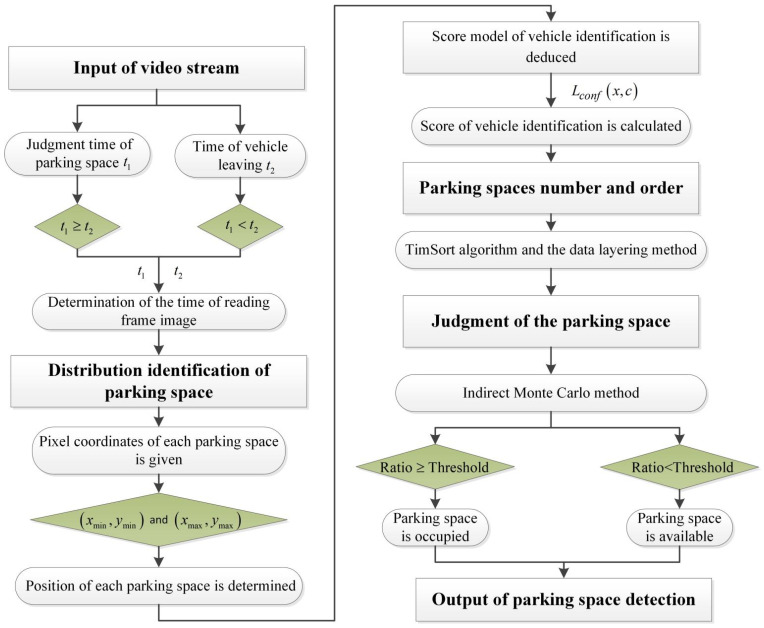
Flowchart of the proposed PSD model.

The proposed method includes four modules (Figure 1): the optimal reading of the video stream, the distribution identification of parking spaces, the numbering and ordering of parking spaces, and the judgment of parking spaces.

### 2.1. Optimal Reading of the Video Stream

The first step in detecting parking spaces using a camera is to read the video stream. OpenCV was employed to read the images from a video stream. However, not every image frame is useful for identifying parking spaces in the process of reading images from a video. To reduce the number of extracted image frames, we did not need to extract every image frame for each second of the video stream. Therefore, the optimal time interval for reading a video needed to be determined. Assuming that the judgment time of identification for each parking space is *T* and that the number of parking spaces covered by the video area is *c*, then the total judgment time can be rewritten as:

(1) $$t_{1}=c\times T.$$

Assume that the time length for a vehicle from its arrival to departure is *t*_2_. On the one hand, the time interval for reading a video should reflect the change in the parking space from the vehicle arriving to the vehicle driving away. On the other hand, the time interval should ensure that all parking spaces have been successfully identified. With regard to the magnitude relationship between *t*_1_ and *t*_2_, two different cases were considered. First, if $t_{1}\geq t_{2}$, then to ensure that all parking spaces could be accurately identified in each image frame, the time interval for reading the video can be determined as *t*_1_. Second, if $t_{1}<t_{2}$, then to ensure that the time interval for reading the video stream reflects the change in the parking space when a vehicle arrives and leaves, the time interval for reading the video can be determined as *t*_2_.

### 2.2. Vehicle Identification Based on the EfficientDet Model

The vacant condition of a parking space is primarily determined by the vehicles parked on it. In the current study, we detected a parking space by identifying the vehicle on it. 

Object detection network models trained via deep learning [27,28,29] include Cascade R-CNN [30], SpineNet [31], CenterNet [32], and EfficientDet [33]. These structures require a backbone network to extract features, such as DLA-34 [34], ResNet [35], InceptionV3 [36,37], Inception ResNet [38], and Efficientnet [39]. TensorFlow denotes the deep learning framework for calculations in the form of computational graphs [40]. All calculations are transformed into nodes on a computational graph. The application programming interface (API) project [37] provides a variety of network structures trained by the Common Objects in Context (COCO) database. Therefore, separately constructing a framework for Cascade R-CNN, RetinaNet, CenterNet, and EfficientDet is unnecessary, and the object detection model can be trained by modifying relevant parameters on this basis. The COCO mAP in Table 1 indicates the detection performance of the models measured using the standard mean average precision (mAP) [41] on the COCO dataset. The higher the mAP value, the better the detection performance.

EfficientDet is a fast and efficient detection method that was proposed by Mingxing Tan et al. [33]; they also proposed a bidirectional feature pyramid network (BiFPN) that allows highly effective cross-scale connections and bidirectional weighted feature fusion. BiFPN removes some nodes; these nodes have only one input and no feature fusion. Moreover, an edge is connected between the original input and output nodes of the same level, as this edge can fuse more features. During the training of the EfficientDet model, top-down and bottom-up bidirectional feature fusion are applied iteratively. The fused features are fed into the class and box networks to produce predictions for class and bounding boxes, respectively. Meanwhile, the weights of the class and box networks are shared across all the features.

We compared the EfficientDet detection framework with other object detection structures such as Cascade-RCNN, RetinaNet, and CenterNet. All the models were applied on a computer with a hardware configuration of a Nvidia 2080Ti 11 GB memory GPU with an Ubuntu 20.04 system. Table 1 clearly indicates that the EfficientDet detection framework was significantly more efficient and accurate than the other structures when dealing with various targets [37]. Meanwhile, considering the rapid detection of parking spaces and the accurate identification of vehicles, we selected EfficientDet-D3 as the detection framework. The trained vehicle identification network model was used to identify the frame image, and the score for vehicle identification using this model was obtained by:

(2) $$\begin{array}{c} L_{conf}\left(x,c\right)=-\sum_{i\in Pos}^{N}x_{ij}^{p}\log\left({\hat{c}}_{i}^{p}\right)-\sum_{i\in Neg}\log\left({\hat{c}}_{i}^{0}\right)\\ {\hat{c}}_{i}^{p}=\frac{\exp(c_{i}^{p})}{\sum_{p}\exp(c_{i}^{p})} \end{array}$$

where $x_{ij}^{p}=\left\{1,0\right\}$ is an indicator for matching the i-th prior box to the j-th ground truth box of category p and $c_{i}^{p}$ is the predicted value of the category confidence of the i-th prior box. 

### 2.3. Detection of Parking Spaces

#### 2.3.1. Determination of Parking Space Position

**Figure 2:**
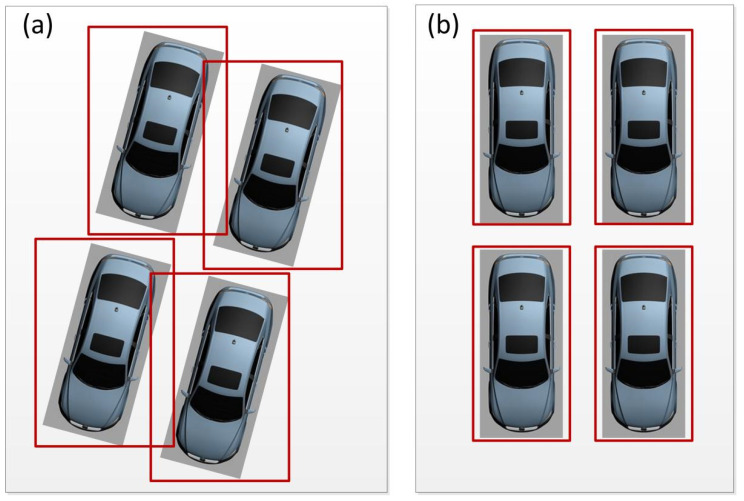
Bounding box of each parking space.

To obtain the distribution of all the parking spaces in a parking lot, the location of each parking space should be determined. The key to determining the position of a parking space is to obtain the coordinate of each parking space. To ensure that the position of each parking space can be obtained, the detection model of the trained object is used to produce the bounding box of each parking space, and the orientation of a vehicle on an image frame extracted from a video stream has two cases—as illustrated in Figure 2. Figure 2a shows that the area of the parking spaces is not a rectangle in the captured images because of the camera angle. The bounding boxes still cover each vehicle, although the dimensions of these boxes are larger than those in Figure 2b.

**Figure 3:**
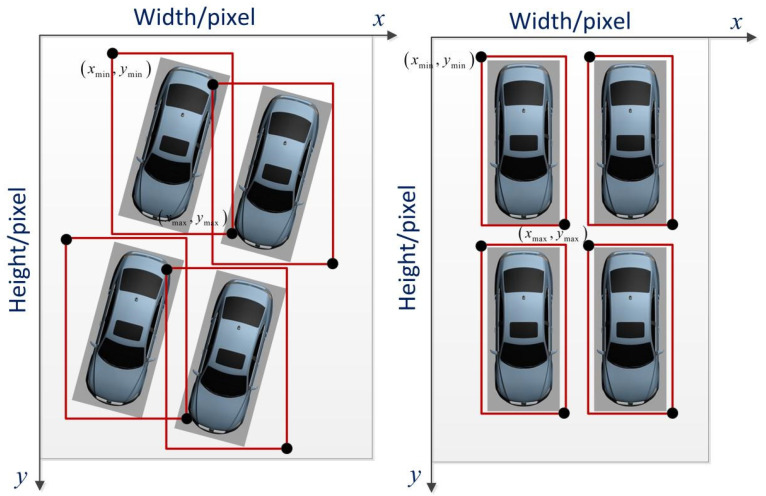
Position and dimensions of each parking space.

The intersection of the left and top sides of a vehicle image frame is the origin of the pixel coordinate system. Thus, the position of each parking space is obtained in accordance with the $\left(x_{\min},y_{\min}\right)$ and $\left(x_{\max},y_{\max}\right)$ of each bounding box on the pixel coordinate system, as shown in Figure 3. In accordance with the position of each vehicle, the distribution of the parking spaces is determined.

#### 2.3.2. Order and Number of Parking Spaces

Sorted and numbered parking spaces are convenient for drivers to use. To sort and number the parking spaces accurately, this study combined the TimSort algorithm and the data layering method.

**Figure 4:**
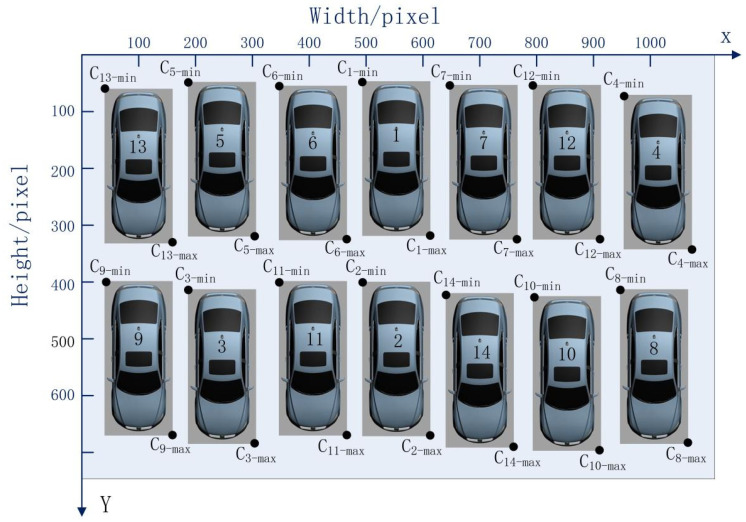
Random parking space numbers.

A parking space is considered in two dimensions—the vertical and horizontal directions—in the process of vehicle identification via deep learning. Simultaneously, the vehicles in parking spaces are not completely in order in real scenarios, as shown in Figure 4.

The positions of the upper left and lower right corners of each parking space were defined as $C_{i-\min}\left(x_{\min},y_{\min}\right)$i=1,⋯,14 and $C_{i-\max}\left(x_{\max},y_{\max}\right)$ i=1,⋯,14. The random parking space numbers were produced as illustrated in Table 2. 

The TimSort algorithm was used to sort all parking space numbers in accordance with the vertical coordinates of each bounding box in each row, as shown in Table 3. 

**Figure 5:**
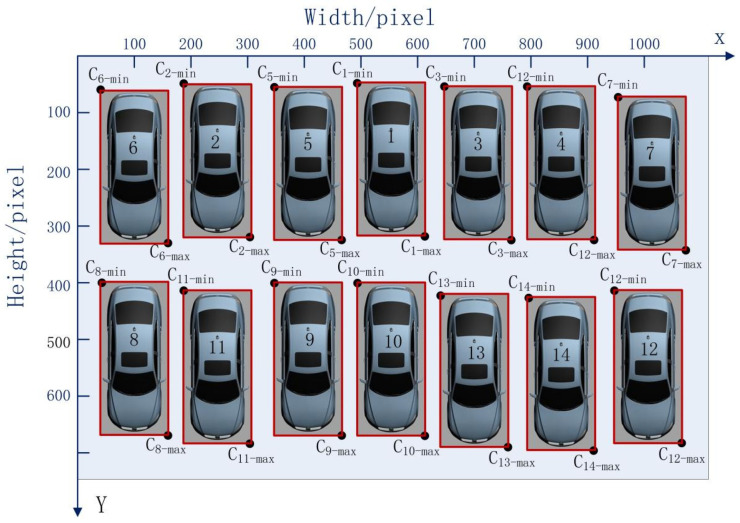
Each vehicle number after sorting with the TimSort algorithm.

In accordance with the position of each vehicle after sorting with the TimSort algorithm, each vehicle number was determined as shown in Figure 5.

Given the difference in the length and width of various types of vehicles and the relative positional difference between a vehicle and a parking space, guaranteeing that the *y*_min_ of each bounding box will be the same is difficult. Therefore, each parking space number was confusing, as shown in Figure 5. To address this issue, the data layering method was used to layer the vertical direction using Equation (3):

(3) $$Data_{}layer=\{i:\left|y_{\min}^{A}-y_{\min}^{B}\right|<q,A,B\in i,A\neq B;\},$$

where *q* is considered the threshold value, which is defined by Equation (4).

In general, *q* is expressed as one-third of the vertical length of the parking lot.

(4) $$q=\frac{1}{3n}\sum_{n}\left(y_{\max}^{j}-y_{\min}^{j}\right)$$

Assume that the difference between the parking spaces of Vehicles A and B is *e*. If e<q, then the parking spaces of Vehicles A and B belong to the same row. Otherwise, they belong to different rows and must be layered. 

In accordance with the data layering method, the order and number of the parking spaces were implemented as shown in Table 4. 

**Figure 6:**
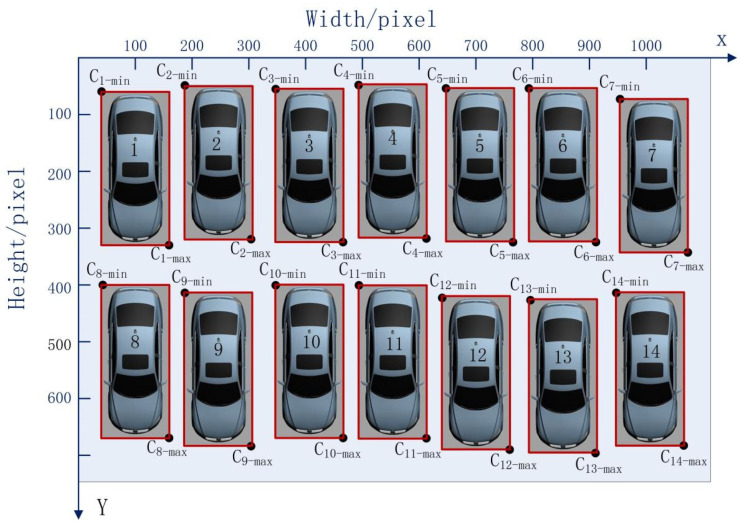
Each vehicle number after sorting with the data layering method.

Table 4 indicates that the data layering method divided the data into two layers—namely, layers 0 and 1. In accordance with the position of each vehicle after sorting with the data layering method, each vehicle number was determined as shown in Figure 6. Ultimately, we were able to determine the order and number of each parking space. 

#### 2.3.3. Judgment of Parking Space Vacancy

**Figure 7:**
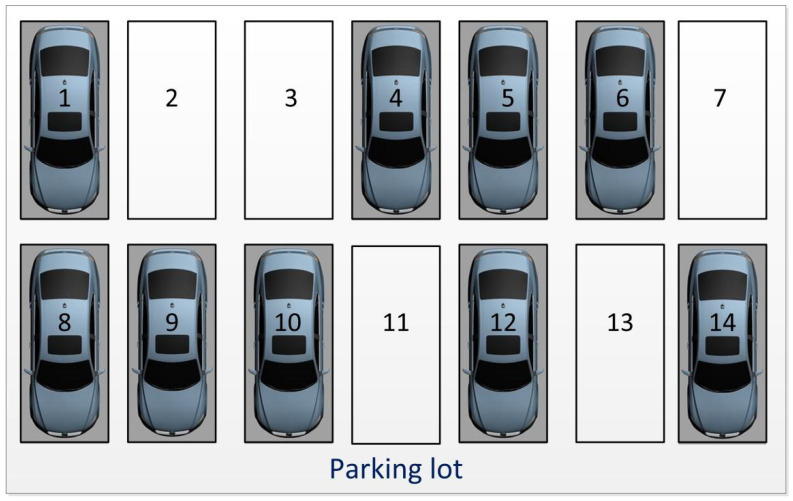
Parking space number.

The judgment of parking space vacancy was determined by the coverage of parking spaces by vehicles. Figure 7 shows one image frame of the parking lot; some parking spaces are clearly occupied, while others are vacant. To judge the vacancy of a parking space, random points were produced within each identified parking space. The range of the number of random points within one square meter was denoted as:

(5) a≤n≤b,

where *a* and *b* represent the lower and upper limits, respectively. Meanwhile, the area of each identified parking space was calculated as follows:

(6) $$S=\left(x_{\max}-x_{\min}\right)\left(y_{\max}-y_{\min}\right).$$

Then, the range of the number of random points within each identified parking space was written as:

(7) Sa≤n≤Sb.

Here, we only considered the upper limit. Ultimately, the number of random points within each identified parking space was Sb.

**Figure 8:**
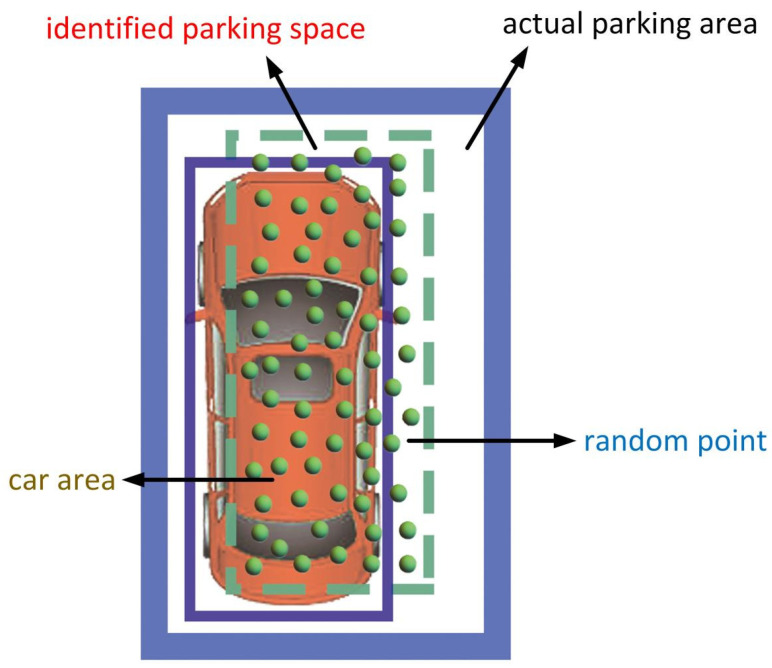
Distribution of one vehicle in a parking space.

The judgment of the coverage of a parking space was largely based on the ratio of random points covered by a vehicle in the parking space, as depicted in Figure 8. The random points covered by a vehicle can be detected using the density clustering model [42]. The indirect Monte Carlo method [43] was utilized to construct the probability discriminant model to judge the parking space.

**Figure 9:**
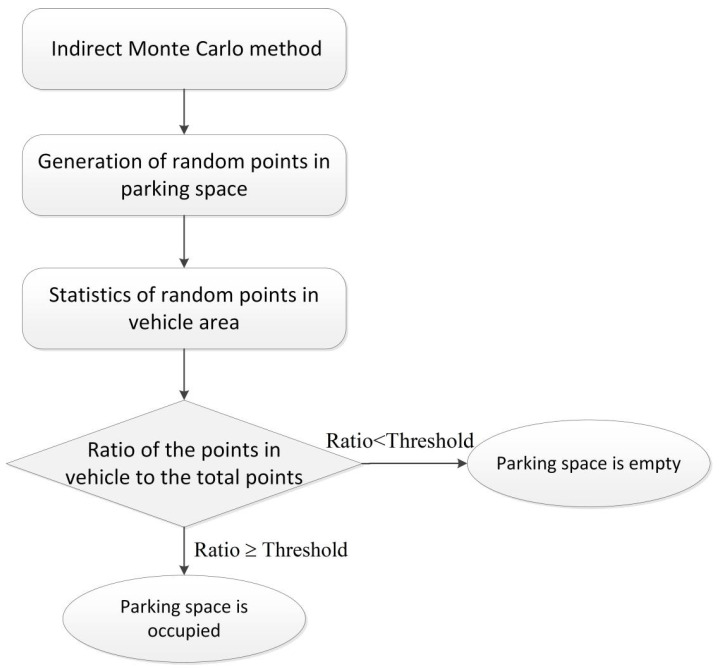
Flowchart for the judgment of the parking space module.

The distribution of one vehicle in a parking space was determined as shown in Figure 8, which includes the vehicle and parking space frame. Figure 9 presents the flowchart for the judgment of the parking space. The ratio of the points in the vehicle to the total points was calculated and compared with the threshold. If Ratio≥Threshold, then the parking space was considered occupied. If Ratio<Threshold, then the parking space was considered vacant.

**Figure 10:**
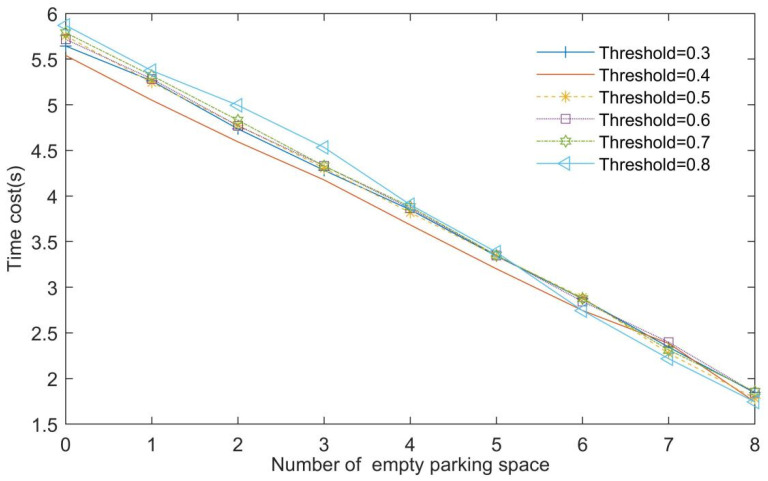
Time cost for different numbers of empty parking spaces.

To determine the threshold value, the time cost of parking space identification for different numbers of empty parking spaces with various thresholds was obtained as shown in Figure 10. For fewer empty parking spaces, more time was clearly taken. However, PSD takes at least 6 s to complete; therefore, the influence of the number of empty parking spaces on the operation time was substantial, and hence, must be considered. Simultaneously, the time costs of the different thresholds were nearly the same. Therefore, we should not consider the influence of different thresholds on operation time. Ultimately, the threshold was determined as 0.8.

**Figure 11:**
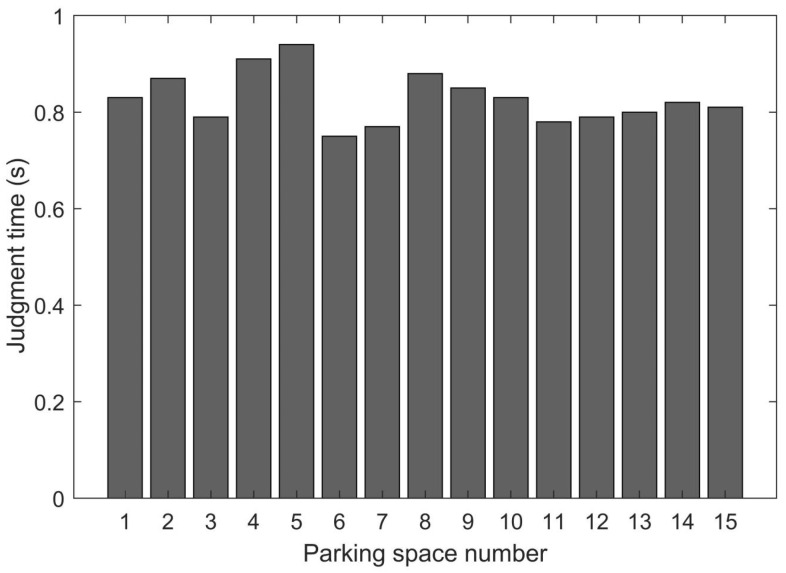
Judgment time for each parking space.

In accordance with the indirect Monte Carlo method, the judgment time T of each parking space could be determined. In particular, a statistical method was used to determine the judgment time of each parking space. With regard to the geometrical relationship between the parking lot and the camera, two different scenarios were considered. The first scenario was as follows: the camera was installed in a low position, such that the distance between the parking lot and the camera was shorter. Meanwhile, the second scenario was as follows: the camera was installed in a high position, such that the distance between the parking lot and the camera was longer. For simplicity, the former is called the short distance scenario and the latter is called the long distance scenario. We selected 15 parking spaces with different positions to calculate the judgment time of a parking space, as shown in Figure 11. 

In accordance with Equation (1), the judgment time for all parking spaces can be calculated when determining the number of parking spaces. As shown in Figure 11, the judgment time T of each parking space was within the range of 0.7 and 0.9, with a mean of 0.8 s. Assuming that 10 parking spaces are present; in accordance with Equation (1), the total judgment time is $t_{1}=8$ s. In accordance with our investigation of 53 drivers, the longest and shortest times for a vehicle to arrive and drive away were 23 s and 7 s, respectively, and the average time was 15 s. Therefore, $t_{2}$ was set as 15 s in the current study. In consideration of $t_{1}<t_{2}$, the time interval for extracting image frames was determined to be 15 s.

**Figure 12:**
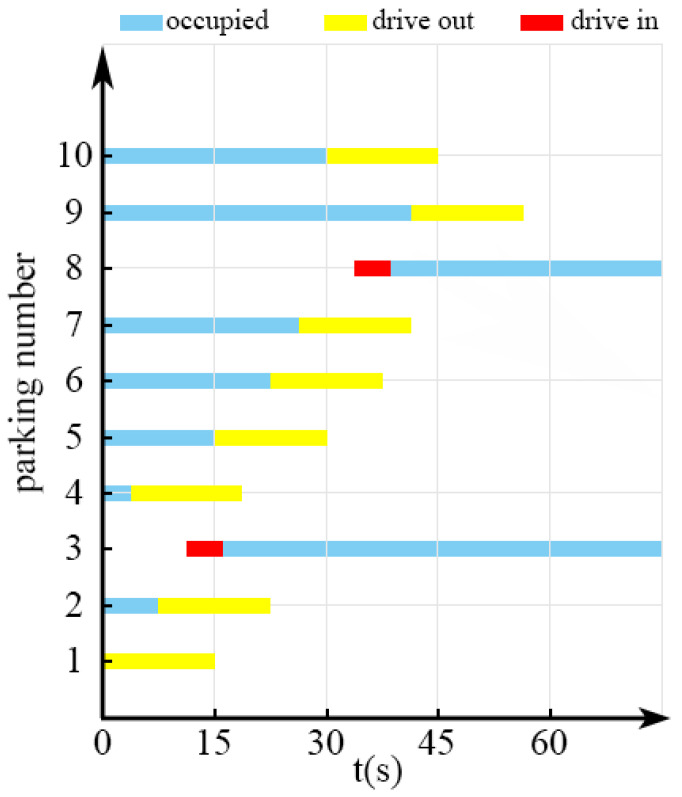
Identification results for all the vehicles entering and leaving the parking spaces.

Figure 12 presents the identification results for all 10 vehicles going in and out of the parking spaces over a period of 75 s. Within the first 15 s, the vehicle in parking space 1 left and was identified. Two vehicles left from parking spaces 1 and 4 at different times. Meanwhile, another vehicle entered parking space 3 at another time. Detecting the change information for parking spaces 2, 3, and 4 within the first 15 s is impractical; however, the vehicles entering and leaving parking spaces 2, 3, and 4 could be identified within 15 s, as shown in Figure 12.

## 3. Experimental Results and Discussion

### 3.1. Data Set Production

**Figure 13:**
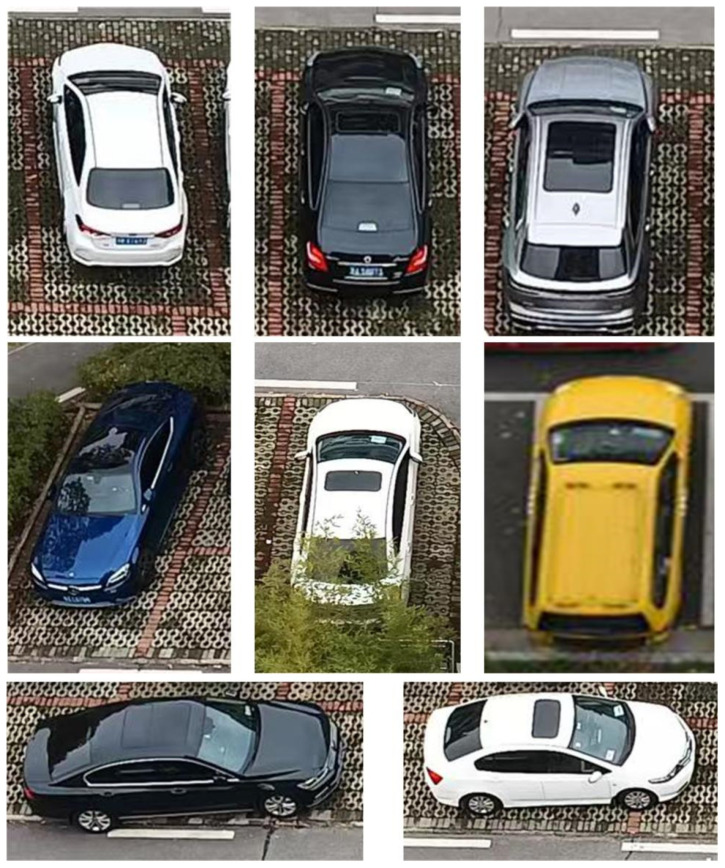
Partial vehicle images in the data set.

In order to verify the proposed method for common use under different conditions, images of different types of vehicles were applied in this experiment, as shown in Figure 13. The 6387 vehicles images were used as training sets and 1146 images were used as verification sets. The 7533 images were manually annotated using the LabelImg tool, and the annotation files generated by each image were converted to TensorFlow’s unified TFRecord data format via a Python script file.

### 3.2. Detection of Vehicles

According to the characteristics of the data set, the parameters in the configuration file corresponding to the pre-training model were adjusted, including the number of categories, the batch size, the initial learning rate, and the related data reading path. The vehicle identification model was not influenced by the variation of vehicle type. A part of the data was selected to obtain the prediction value using the CNN algorithm at the beginning of the iteration. The amount of this data was the batch size. According to the size of the data set and the computer configuration, the batch size of the initial training was selected first, and then it was adjusted according to the variation in the loss function value and the identification effect.

The vehicle identification model was trained using a standard gradient descent algorithm. TensorFlow_Slim is a lightweight library for defining, training, and evaluating complex models in TensorFlow. In the process of model training, TensorFlow_Slim provides a simple but very powerful set of training models. The return value of the loss function during training is the value of the objective function generated in the process of each iteration. In other words, the sum of the localization loss and the confidence loss are an indicator for measuring the performance of the prediction model.

To debug and optimize the training process of the neural network, TensorFlow provides a visualization tool—Tensor Board—which monitors and displays the training process by reading the recorded data files. Within reason, the larger the batch size, the higher the memory utilization rate and the faster the data is processed. In training, the batch size was set to 16 when using the single NVIDIA GeForce RTX 2080Ti GPU.

**Figure 14:**
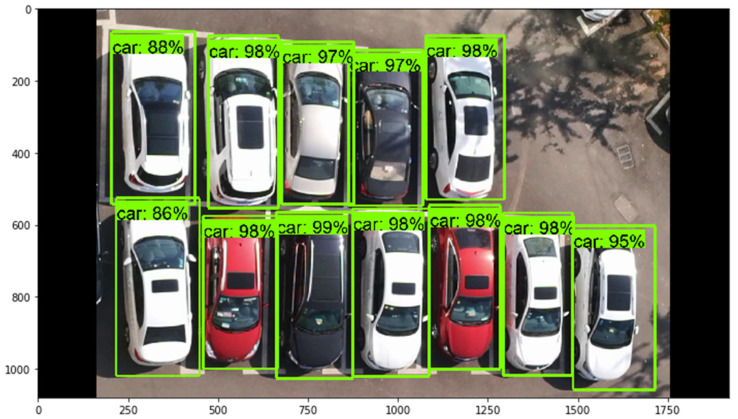
Identification effect of vehicles using the training model.

The accuracy of the verification set was evaluated after executing the test script, as shown in Figure 14. Figure 14 shows the evaluation and identification effect in the IMAGES panel. The higher the identification scores of the vehicle, the better the identification effect. The advantage of the EfficientDet detection framework was that it had higher accuracy when detecting targets and had fewer parameters than the other structures. According to Figure 14, the identification effects of the vehicles were all greater than 85%.

### 3.3. Detection of Parking Spaces

**Figure 15:**
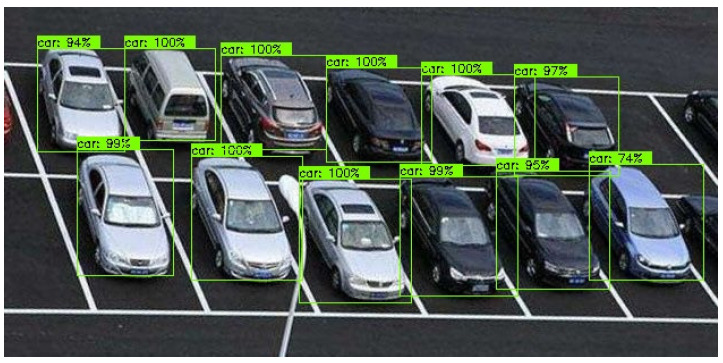
Identification of vehicles under the condition of full parking.

In this process of the video stream reading, the average judgment time for each empty parking space was $T=0.5^{\prime }$ and the number of parking spaces covered by the video area was *c*. According to Equation (1), the judgment time was 0.5c. Assume the length of time taken by the vehicle from the start to leaving the parking space was $0^{\prime }$; the reading time of video stream was equal to 0.5c. The trained identification model was applied to produce bounding boxes for each vehicle and the score of each vehicle was obtained, as shown in Figure 15.

**Figure 16:**
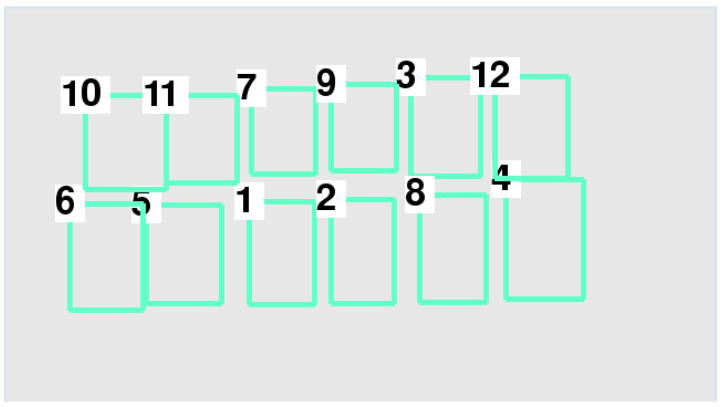
Random number of each parking space.

According to the score of each vehicle, we confirmed that every parking space was covered by a vehicle. Then, the random number of each parking space was obtained, as shown in Figure 16.

**Figure 17:**
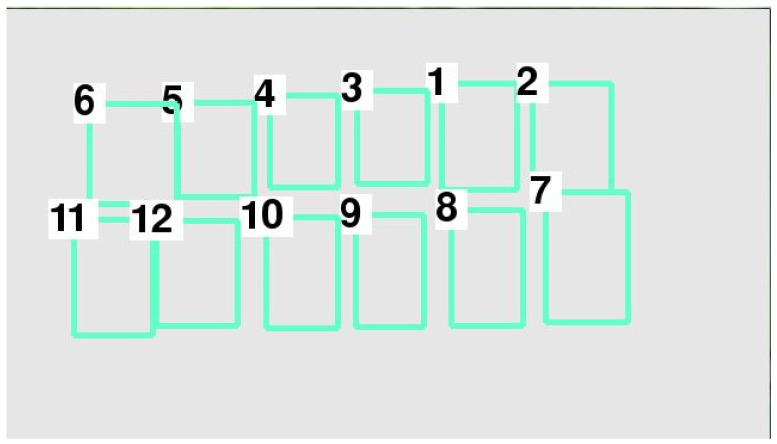
Parking space numbering by the Timsort algorithm.

**Figure 18:**
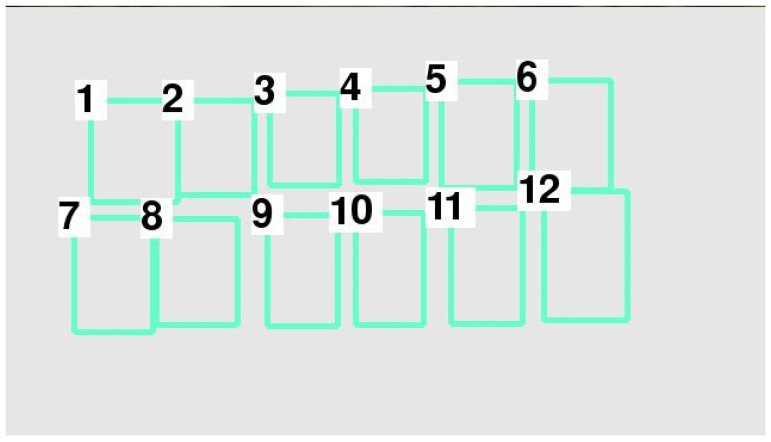
Parking space numbers after data layering.

The $\left(x_{\min},y_{\min}\right)$ and $\left(x_{\max},y_{\max}\right)$ of each bounding box on the Pixel Coordinate System was obtained. As shown in Figure 16, the parking space numbers were disordered. In view of this, the Timsort algorithm was applied to sort the bounding box number of each parking space, as shown in Figure 17. It was clearly visible that the parking space number was in order in the vertical direction, but still in disorder in the horizontal direction. Meanwhile, the $\left(x_{\min},y_{\min}\right)$ and $\left(x_{\max},y_{\max}\right)$ of each bounding box on the Pixel Coordinate System was obtained, as shown in Table 5. As described in Section 2, the data layering method was applied to divide the layer in the vertical direction, and then the Timsort algorithm was applied to sort the parking space numbers at each layer, as shown in Figure 18. It was shown that the parking space numbers were in order. The position of each parking space was obtained, as shown in Table 6.

## 4. Conclusions

In this study, we proposed a method for detecting parking spaces in a parking lot by utilizing deep learning in object identification. The proposed method consisted of the detection of parking spaces and the identification of the distribution of parking spaces. In the PSD process of using the video stream, the optimal reading time interval for the video stream was provided. A vehicle identification model based on EfficientDet-D3 was constructed. To improve the training efficiency of the identification model, we flexibly changed the learning rate during the training of the model. Simultaneously, we proposed the combined methods of the TimSort algorithm and the data layering method to determine the number and order of the parking spaces. We found that the indirect Monte Carlo method could be used to judge parking space occupancy. To evaluate the performance of the proposed method, we conducted the detection of a large parking lot with realistic scenarios. The detection results showed that the proposed method could detect parking spaces efficiently when vehicles came and went.

In the future, we will focus on parking space assignment and guidance. In consideration of one parking lot, if the number of requests for a parking space service is higher than the total number of empty parking spots, then the system will inform the users to choose other nearby parking lots with certain numbers of available parking spaces and inform the users the distances to those parking lots. Meanwhile, on the basis of the occupation time of each parking space and the allowed maximum parking period, the system can inform users how long they will need to wait for the next available empty space. On the basis of such information, the users can make a decision on whether they should wait for a parking space or drive to the closest parking lot with available parking spaces.

## Figures and Tables

**Table 1:** COCO-trained partial models.

Model Name	Speed/ms	COCO mAP
Cascade R-CNN_ResNet-101	410	42.8
CenterNet_DLA-34	31	41.6
RetinaNet_ResNet-101	32	39.9
EfficientDet-D1	16	40.5
EfficientDet-D3	37	45.6
EfficientDet-D7x	285	55.1

**Table 2:** Positions of random vehicle numbers.

	1	2	3	……	12	13	14
*x*_min_	493.33	493.92	187.25	……	793.33	40.58	640.58
*y*_min_	48.00	400.58	413.92	……	53.92	59.42	422.75
*x*_max_	612.75	612.75	303.92	……	911.42	159.42	759.42
*y*_max_	318.25	670.00	683.92	……	324.58	330.00	690.00

**Table 3:** Position of each vehicle after sorting using the TimSort algorithm.

	1	2	3	……	12	13	14
*x*_min_	493.33	187.25	647.25	……	947.25	640.58	796.08
*y*_min_	48.00	48.58	53.92	……	413.92	422.75	426.83
*x*_max_	612.75	304.00	765.33	……	1066.08	759.42	910.58
*y*_max_	318.25	319.42	324.75	……	682.75	690.00	696.08

**Table 4:** Position of each vehicle after sorting using the data layering method.

	1	2	3	……	12	13	14
*x*_min_	40.58	187.25	347.25	……	640.58	796.08	947.25
*y*_min_	59.42	48.58	55.08	……	422.75	426.83	413.92
*x*_max_	159.42	304.00	465.92	……	759.42	910.58	1066.08
*y*_max_	330.00	319.42	324.58	……	690.00	696.08	682.75
Data layer	0	0	0	……	1	1	1

**Table 5:** Position of the vehicles after sorting and the numbering of the parking spaces by the Timsort algorithm.

Parking Number	1	2	3	4	5	6	7	8	9	10	11	12
*y*_min_	186.7453	186.3284	204.6493	216.4648	235.2376	237.4085	460.5099	504.3114	516.6836	523.6484	530.2592	532.6093
*x*_min_	1096.534	1324.633	882.5272	663.6877	433.7274	212.866	1356.459	1121.151	879.7378	655.9752	172.1902	380.7485
*y*_max_	453.757	460.8913	440.2206	448.5158	472.4123	490.6271	787.125	797.4492	798.6421	801.9327	820.0494	797.8188
*x*_max_	1286.16	1522.195	1059.158	836.6179	626.4663	431.8626	1564.168	1299.886	1052.452	835.4104	370.5828	583.7634
Scores	0.96967	0.815933	0.925088	0.928011	0.907685	0.926269	0.962921	0.928651	0.989354	0.991868	0.938664	0.954729

**Table 6:** Position of the vehicles after sorting and the numbering of the parking spaces by the data layering method.

Parking Number	1	2	3	4	5	6	7	8	9	10	11	12
*y*_min_	237.4085	235.2376	216.4648	204.6493	186.7453	186.3284	530.2592	532.6093	523.6484	516.6836	504.3114	460.5099
*x*_min_	212.866	433.7274	663.6877	882.5272	1096.534	1324.633	172.1902	380.7485	655.9752	879.7378	1121.151	1356.459
*y*_max_	490.6271	472.4123	448.5158	440.2206	453.757	460.8913	820.0494	797.8188	801.9327	798.6421	797.4492	787.125
*x*_max_	431.8626	626.4663	836.6179	1059.158	1286.16	1522.195	370.5828	583.7634	835.4104	1052.452	1299.886	1564.168
Scores	0.926269	0.907685	0.928011	0.925088	0.96967	0.815933	0.938664	0.954729	0.991868	0.989354	0.928651	0.962921
Data layer	0	0	0	0	0	0	1	1	1	1	1	1

## Data Availability

Not applicable.
